# Acute Invasive Fungal Rhinosinusitis: Frozen Section Histomorphology and Diagnosis with PAS Stain

**DOI:** 10.1007/s12105-018-0965-8

**Published:** 2018-09-12

**Authors:** Henry Crist, Max Hennessy, Jacob Hodos, Johnathan McGinn, Bartholomew White, Sakeena Payne, Joshua I. Warrick

**Affiliations:** 1 Department of Pathology, College of Medicine, The Pennsylvania State University, 500 University Drive, Hershey, PA 17033-0850 USA; 2 Division of Otolaryngology – Head and Neck Surgery, Department of Surgery, College of Medicine, The Pennsylvania State University, Hershey, PA USA

**Keywords:** Acute invasive fungal rhinosinusitis, Invasive fungal sinusitis, Frozen section, Diagnosis, Fungus stain, PASF

## Abstract

Acute invasive fungal rhinosinusitis (AIFRS) is a fulminant infection in immunocompromised patients requiring rapid diagnosis (DX), frequently made on frozen section (FS) of sinonasal biopsies, followed by prompt surgical debridement. However, FS interpretation is often difficult and DX sometimes not possible. In this study we sought to characterize reasons for misinterpretation and methods to improve diagnostic accuracy. The FS slides from 271 biopsies of suspected AIFRS in a 16-year period were reviewed and the morphologic features evaluated for their utility in DX. Recurring specific patterns of necrosis were identified, which to our knowledge have not been described in the literature. Although they provide strong evidence for AIFRS, identifying fungus consistently in necrotic tissue is essential for DX. Clues to identifying fungus and pitfalls in misidentification were identified, but even with expert knowledge of these, a gap in accurate DX remained. The key to FS DX of AIFRS is to improve fungus identification in necrotic tissues. Methods had been sought in the past to stain fungus at FS without consistent success. The Periodic Acid Schiff’s Reaction for Fungi was modified by our histopathology department for use on frozen tissue (PASF-fs) resulting in effective staining of the fungus. It stained fungus on all 62 positive slides when applied retrospectively over hematoxylin and eosin (H&E) stained FSs and used prospectively at FS for DX. Although knowledge of histologic morphology on FS is important, the crucial value of this study is the novel use of PASF-fs to identify fungus in the DX of AIFRS.

**Electronic supplementary material:**

The online version of this article (10.1007/s12105-018-0965-8) contains supplementary material, which is available to authorized users.

## Introduction

Acute invasive fungal rhinosinusitis (AIFRS) is typically a fulminant infection with high morbidity and mortality [1, 2] in immunocompromised patients [3]. Mortality rates can be reduced with immediate treatment, typically involving aggressive surgical resection of infected tissue, support for immune system recovery, and systemic antifungal therapy [3–7]. Thus, a rapid and accurate diagnosis (DX) is crucial in providing timely and effective treatment. Histopathologic analysis by frozen section (FS) has become a preferred diagnostic method owing to its rapid turnaround in the DX of AIFRS [8–11] but its accuracy is diminished by difficulties in recognizing the disease. We theorize this may be in part because a detailed histomorphologic description of this disease process as seen on FS has not been documented in the literature. In this study, we present a description of the important diagnostic features of AIFRS with emphasis on problems in interpretation at FS and potential solutions.

## Materials and Methods

This study had three main aims: (1) to identify and describe the pathology and specific histologic features of AIFRS and to evaluate their diagnostic utility, (2) to identify possible solutions to problems and potential aids in the DX, and (3) to consider alternatives to FS for the DX. This study was performed under approval of the Pennsylvania State College of Medicine Institutional Review Board. We retrospectively collected all sinonasal biopsies sent for FS with clinical suspicion for AIFRS at our institution from 2002 to 2017. A senior head and neck pathologist (HSC) reviewed all FS slides, blinded to the original FS and final pathologic diagnoses.

Slide review entailed documenting the reviewer’s opinion of best FS DX and the presence of specific histologic features in AIFRS. The final pathologic DX was confirmed on review of the routinely processed hematoxylin and eosin (H&E) slides and Grocott–Gomori’s methenamine silver (GMS) stained slides on all specimens. All slides with a positive DX were re-reviewed by a second blinded head and neck pathologist (JIW) to confirm the DX and presence of the specific histologic features. Any discordance between the review pathologist and the final pathologic diagnoses, original FS diagnoses, or between the review pathologists was re-reviewed by the two pathologists using a double-headed microscope to reach a final consensus FS DX. Routinely processed H&E and GMS stained slides were then reviewed for accuracy of the final FS DX with the final pathologic DX which was treated as the true DX.

In this study, each biopsy (BX) for FS, irrespective of other biopsies accessioned from the same or separate surgical procedures, was a subject in assessment of its histomorphology and evaluation for features of utility in DX. The FS diagnoses were rendered by pathologists of diverse subspecialties assigned to the service on a rotational basis with 90% performed by the pathologist on call nights or weekends, some not experienced in the pathology of the disease. Difficult cases occasionally were given non-definitive diagnoses. In our study we defined a DX providing support for surgery, ‘suspicious or compatible with’, as positive (POS) and one not stating support, ‘cannot rule out or deferred’, as negative (NEG). Sensitivities were calculated for the original FS DX, for the reviewed diagnoses, and for the specific histologic features useful for DX. A Periodic Acid Schiff’s Reaction stain for fungus was modified for use on FSs (Supplemental Online Resource File), and its ability to stain fungus on FS slides was demonstrated by applying it over the H&E stain after removing the coverslip from one of the two original slides routinely prepared for FS. Subsequent to this initial retrospective evaluation, the PASF-fs stain was incorporated into our routine intraoperative evaluation of AIFRS. Data collected during the last year of the study represented its prospective utilization in the FS DX.

## Results

During the study period 146 patients had sufficient clinical suspicion to warrant surgical intervention or BX. Sixty-three (43%) were diagnosed with AIFRS while 83 (57%) did not have the disease. The DX was made clinically on 22 (15%) who were treated directly with surgical debridement while the remaining 41 (28%) were diagnosed on BX by FS. Of the patients with AIFRS, 65% then required a BX to make or confirm the DX (Table 1). The study cohort consisted of the 271 biopsies sent for FS with 208 (77%) being NEG and 63 (23%) POS on final pathologic DX. Fifty-one of these 63 biopsies (81%) were diagnosed as POS on H&E at the time of FS. Twelve biopsies from 10 separate patients thus were considered false NEG. The missed diagnoses were caused by the inability to recognize fungus, although sometimes compounded by technical issues compromising the adequacy of the slides. Of the 12 false NEG specimens, 7 were diagnosed POS on initial review of the H&E 58/63 (92%) with an additional POS identified by consensus review 59/63 (94%). Of the four false NEG FSs with no fungus recognized on H&E, three had identifiable fungus when the slides were stained retrospectively with PASF-fs. No fungus was present on the remaining FS slide but the final DX was made on several hyphae identified by GMS on the permanent section at a deeper level in the block. In total, (62/63) 98% of the POS biopsies had fungus identified on a FS slide with the PASF-fs stain. Of the NEG biopsies, (6/208) 3% had a false POS FS DX. All 6 were from patients who had AIFRS with other specimens that were POS. All patients (100%) in the study period who were diagnosed either clinically or on FS had a final pathologic DX confirming AIFRS (Table 2).

**Table 1:** Patients having surgical debridement or biopsy with FS for suspected AIFRS

Total patients	146
Patients negative for the diagnosis of AIFRS	83 (57%)
Patients positive for the diagnosis of AIFRS	63 (28%)
Patients with surgical debridement diagnosed clinically without prior frozen section biopsy	22 (15%)
Patients with surgical debridement following diagnosis by prior frozen section biopsy	41 (28%)
Percentage of patients with AIFRS requiring frozen section biopsy for diagnosis	41/63 (65%)

**Table 2:** Initial FS, review and PASF-fs diagnoses compared to the final pathology (reference) DX

Final pathology diagnosis	Biopsy specimens number (+) or (−) (total n = 271)	Initial H&E frozen section diagnosis	Review H&E frozen section diagnosis	PASF-fs frozen section diagnosis
Positive (+)	(+) 63 (100%)	(+) 51 (81%)	(+) 59 (94%)	(+) 62 (98%)
Positive (+)	(−) 0 (0%)	(−) 12 (19%)	(−) 4 (6%)	(−) 1 (2%)
Negative (−)	(+) 0 (0%)	(+) 6 (3%)	(+) 0 (0%)	(+) 0 (0%)
Negative (−)	(−) 208 (100%)	(−) 202 (97%)	(−) 208 (100%)	(−) 25^a^ (0%)

^a^PASF-fs was done only on the 30 prospective cases (25 negative, 5 positive)

The pathophysiologic process of AIFRS was seen as one of fungus invading tissue causing necrosis, most often by invading and occluding vascular channels. The histopathologic criteria for DX were identified as first, necrosis which was the most sensitive (90%) and reasonably specific in this context (93%). Even in the NEG specimens with necrosis but without identifiable fungus, all 6 were secondary to AIFRS which was diagnosed on separate biopsies. Second, fungus invading stroma which was slightly less sensitive than necrosis (83%), and third, the presence of vascular fungal thrombosis was the least sensitive feature for AIFRS (70%). The necrosis varied from partial to complete with different patterns presenting challenges to interpretation as the necrosis advanced to its extreme stage, becoming unrecognizable as the residue of former tissue. Fungus also became harder to identify as the necrosis progressed, frequently only being surmised by its characteristic outlines or spaces in the necrotic tissue. In our cohort, mucormycosis and Aspergillus were present equally according to the appearance of the fungal morphology, although this could not be confirmed as too few cultures were positive with patients frequently receiving antifungal therapy.

During the prospective period of the study in its last year utilizing the PASF-fs stain for DX, there were 30 specimens collected with the results mirroring those of the larger retrospective cohort in the rates of POS diagnoses, 5/30 (17% vs. 23%); of fungus identified on H&E, 4/5 (80% vs. 81%) and on PASF-fs, 5/5 (100% vs. 98%); and in false NEG diagnoses 1/5 (20% vs. 19%). Although the numbers are small in this prospective group, it was representative also in the fungi present in the large retrospective group, namely Aspergillus, mucormycosis, and an uncommon *Alternaria* sp.

## Discussion

AIFRS is a fulminant, potentially deadly fungal infection affecting immunocompromised patients. This group most often is comprised of those with hematologic malignancies or post bone marrow transplant, which represented 92% of our cohort [1, 12–14]. In published series as many as half the patients with AIFRS died from the disease with progression from initial presentation to death occurring in hours to days [3]. Furthermore, adverse effects of prolonged antifungal therapy such as organ failure, lead to increased patient morbidity and mortality [15]. Therefore, rapid diagnostic evaluation in at-risk patients is vital both for survival and reduction in morbidity from advancing tissue destruction. Prior to frank tissue necrosis, early findings may be subtle and non-specific with the appearance of edematous, dry, or pale mucosa [2]. Given the need for DX at the earliest possible stage, at our institution, BX with FS is performed in suspected patients with minor or non-definitive abnormalities, and when the clinical degree of concern is high, even on endoscopically normal tissue [2]. Biopsies are primarily taken from the sites of visibly abnormal tissue, but the middle turbinate has a higher yield for those with no distinct abnormal findings [2].

**Fig. 1:**
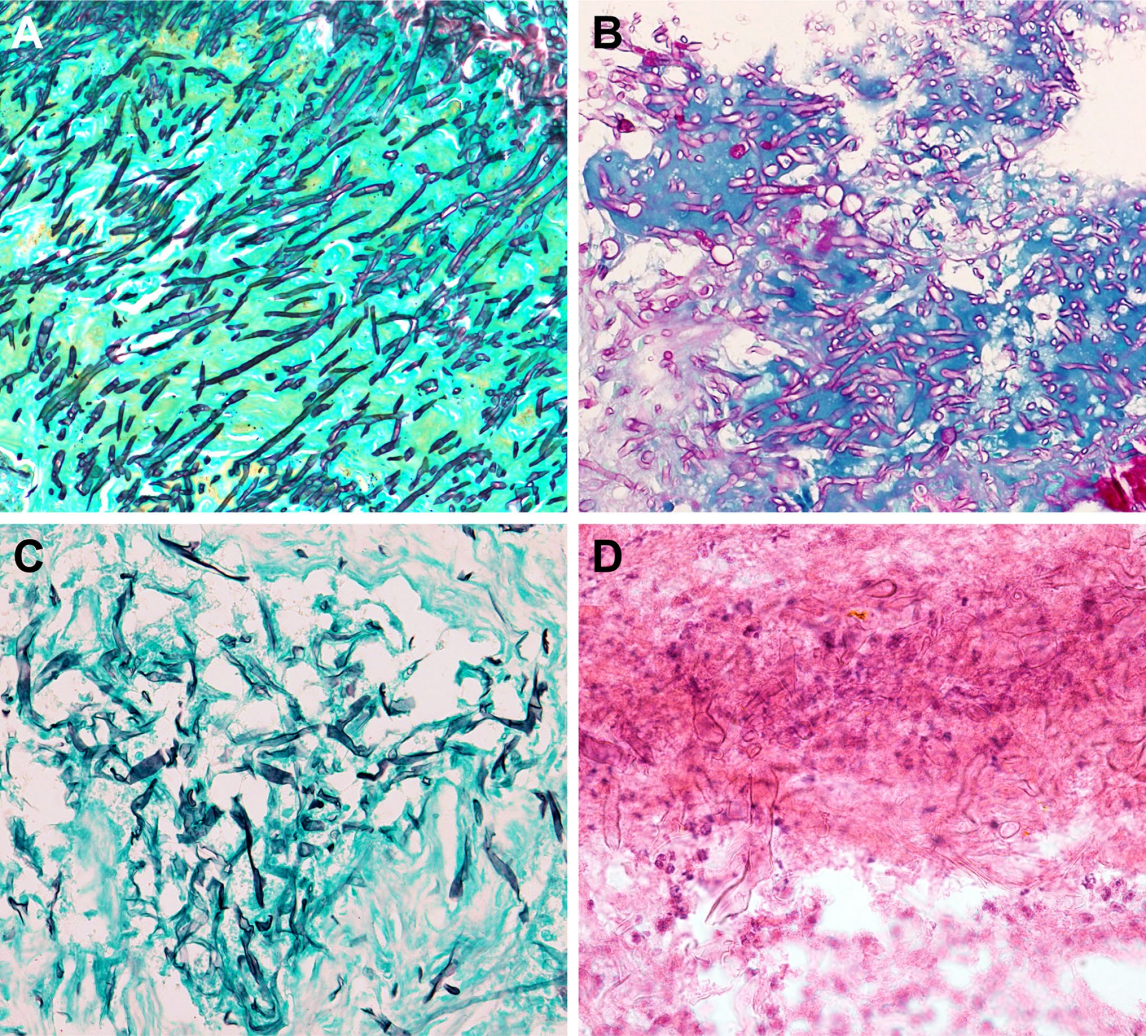
Aspergillus morphology: **a** uniform, septate and regular acute angle dichotomous branching in a progressive arboreal pattern (GMS stain on permanent section × 300), **b** similar hyphae in necrotic tissue in a random pattern with occasional prominent vesicles (PASF-fs stain × 400). Mucormycosis morphology: **c** broad irregularly contoured and pleomorphic hyphae with thin folded twisted ribbon-like shapes (GMS stain on permanent section × 300), **d** similar delicate, poorly stained hyphae (H&E × 500)

The causative fungal organisms in AIFRS have a propensity for angioinvasion as a major component of their pathophysiology. These fungi primarily include the Aspergillus and similar species (grouped together here as ‘Aspergillus’) and the genera Mucor and Rhizopus of the Zygomycetes class, referred to as ‘mucormycosis’ in this paper for simplicity [15]. These two groups of fungi have morphologies which frequently enable them to be differentiated with reasonable accuracy, although culture is necessary to be definitive. In consideration of initial antifungal therapy and prognosis, the clinicians routinely ask if the fungus looks like mucormycosis. The hyphae of Aspergillus typically are uniform, septate and regular with a dichotomous (equal diameter) branching at acute angles in a progressive arboreal pattern, although this pattern is not usually seen in necrotic tissue (Fig. 1a, b). When the hyphae degenerate, they may become swollen, or form prominent vesicles occasionally appearing similar to those of mucormycosis. The hyphae in mucormycosis typically are broad, irregularly contoured and pleomorphic in a haphazard pattern with right angle branches of smaller diameter and only rare true septae. Because of their thin walls, they are often folded, twisted, collapsed, ribbon-like and so delicate they are not visible (Fig. 1c, d). The hyphae may be abundant and tightly packed in necrotic tissue or be few and scattered.

**Fig. 2:**
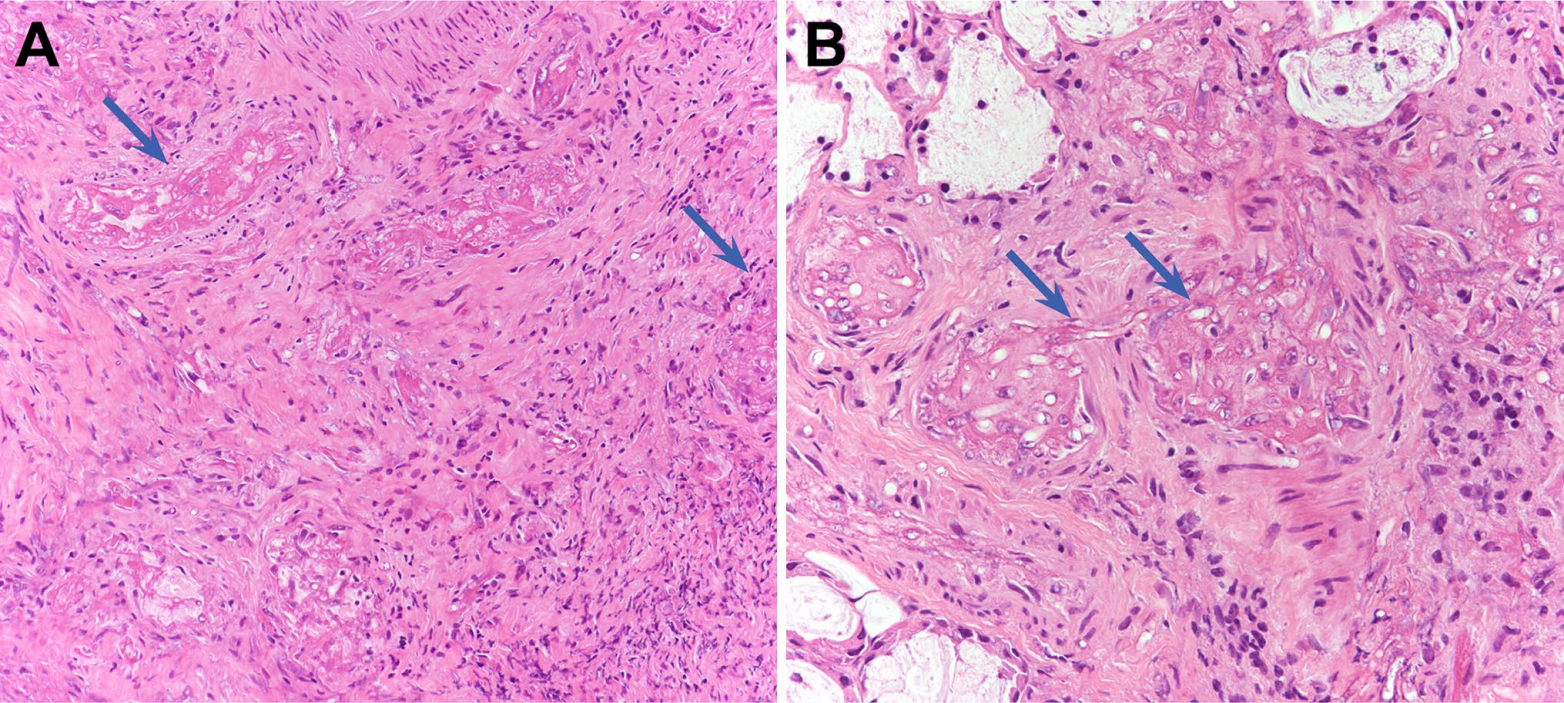
Vascular fungal thrombosis: **a** thrombosis readily evident in dilated vessels with clear spaces indicating fungus (H&E × 200), **b** higher power of vessels on cross-section with non-staining hyphae evident as clear spaces (H&E × 400)

Necrosis, frequently with fungal vascular thrombi is the hallmark of AIFRS [15] (Fig. 2), although its accurate identification is not always straightforward. Occasionally the substance stained on the slides in cases of extreme necroses is unrecognizable. In order to determine what might represent the residue of necrotic tissue, a combination of special stains was applied to selected slides. In addition to H&E, they included GMS, Periodic Acid Schiff’s Reaction, Masson’s trichrome, and phosphotungstic acid haematoxylin. The patterns shown by this combination of stains aided in differentiating what was mucin, hemolyzed blood and fibrin from degenerated fragments of collagen, representing the necrotic remnants of tissue.

**Fig. 3:**
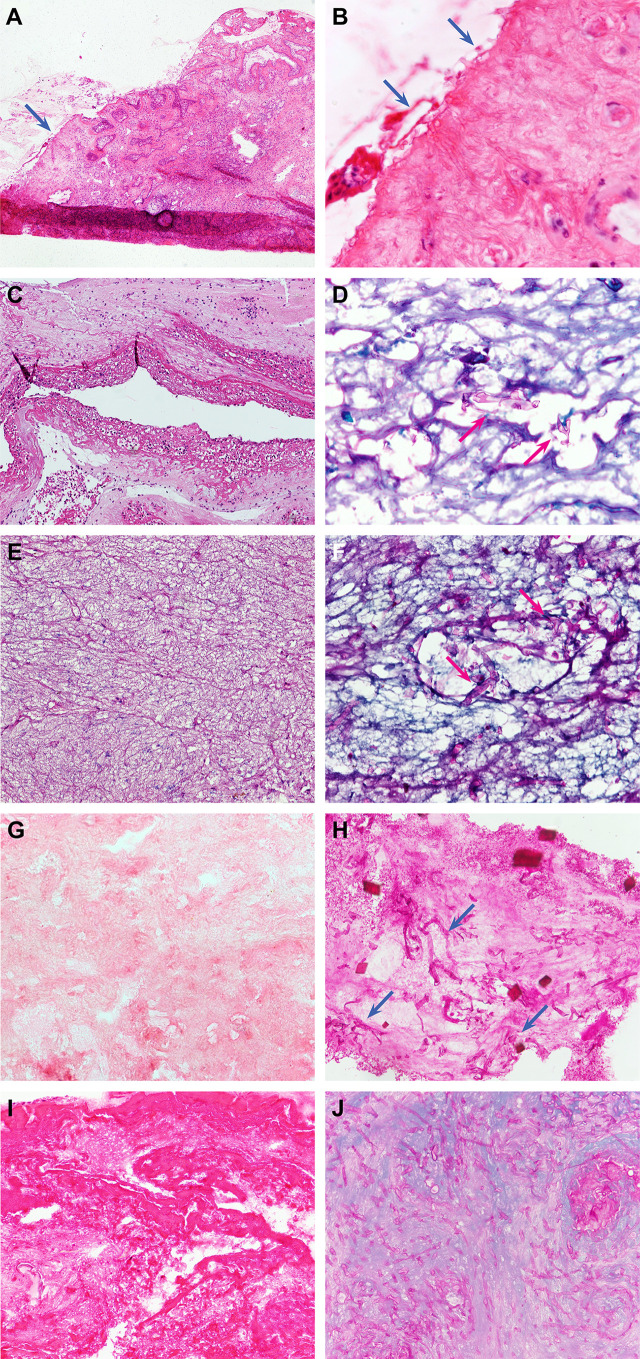
Patterns of necrosis: **a, b** focal superficial erosion: may be tiny and overlooked as in this case although Aspergillus could be identified (H&E × 40 & × 400), **c, d** partial necrosis: evident with outlines of the mucosa but submucosal tissue essentially necrotic. Fungus could not be identified on H&E but mucormycosis was readily seen on PASF-fs (H&E × 100 & PASF-fs stain × 400), **e, f** complete sieve or mesh-like necrosis: there is nothing in this delicate sieve-like pattern that identifies it as tissue although it has the blue staining of collagen on trichrome stain. Fungus could not be identified on H&E but mucormycosis was abundant when stained by PASF-fs (H&E × 300 & PASF-fs × 400), **g, h** Complete thin amorphous necrosis: this thin pale pink staining amorphous material is not recognizable as the remnants of tissue, yet Aspergillus is easily seen on special staining (H&E × 300 & PASF-fs × 400), **i, j** thick amorphous necrosis: neither tissue nor fungus could be identified but Aspergillus was abundant on special stain (H&E × 40 & PASF-fs stain × 400)

Initially, AIFRS can present as a small mucosal erosion. But as the fungus invades blood vessels, necrosis extends deeper beyond the immediate area of invasion. The necrosis may be hemorrhagic or have a thick amorphous appearance. The degree varies from partial, where the underlying tissue architecture and components can be identified in varying degrees, to the complete loss of all structure. In its end stage, when the necrosis is so complete, its remnants may have a sieve or mesh-like network of strands mimicking fibrin, or be so amorphous to be misinterpreted as mucin (Fig. 3). Conversely, mucin admixed with blood and fibrin can mimic necrosis. No explanation was identified for why the necrosis would assume a particular pattern but in this limited number of cases, the sieve or mesh-like and thin amorphous types were more associated with mucormycosis, while those that were dense and thick were present more often with Aspergillus.

**Fig. 4:**
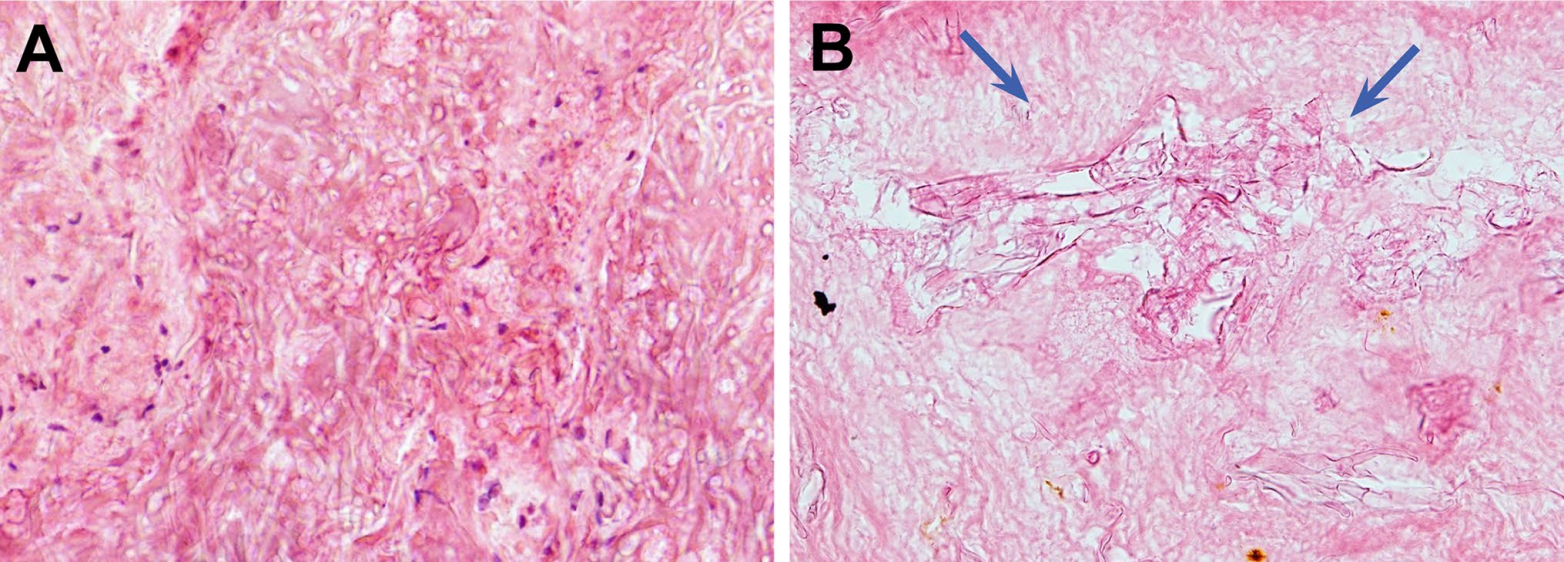
Fungal identification: **a** Aspergillus and **b** mucormycosis can both be recognized by the characteristic shapes of the empty spaces the hyphae occupy. The shapes and sometimes fungal walls can be more evident in refracted light by lowering the microscope condenser, as done for the photograph in **b** (both H&E × 400)

Although, the necrosis occurs in patterns that are somewhat characteristic in AIFRS, alone these patterns are not specific. They can be seen following antifungal therapy when viable fungus is no longer present for the DX and in unrelated pathology such as nasal septal perforation, vasculitis, acute rhinosinusitis or infarcted polyps. However the presence of a characteristic pattern of necrosis with its high specificity in the appropriate clinical setting without other apparent cause, could be considered a presumptive DX of AIFRS, or at least raise high concern for its presence. Fungus is more likely to be seen within thrombosed vessels, at the edges of the BX or in thinner areas, but staining of their walls is frequently lost in the presence of necrosis requiring interpretation from the shapes of empty spaces they occupy or by seeing their unstained walls in refracted light when the microscope condenser is lowered (Fig. 4). Tissue elements seen in necrosis such as collagen, fibrin, mucus strands, vascular channels, slits, small cracks, folds in the tissue, and even keratin or folded epithelial cell walls may mimic fungal elements or the spaces they occupy leading to a false POS interpretation (Supplemental Fig. 1).

**Fig. 5:**
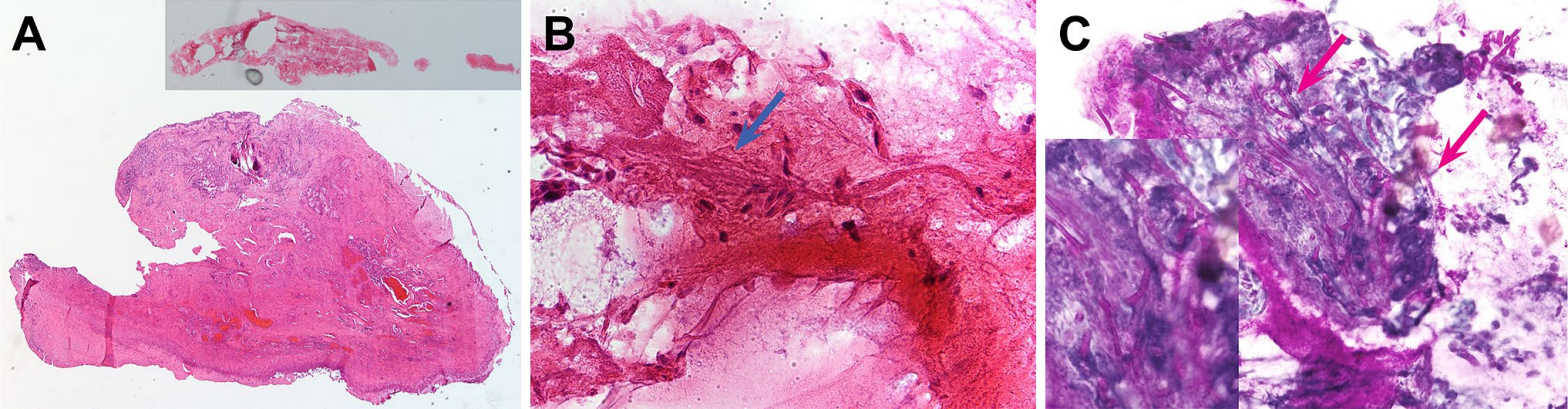
Technical pitfalls: **a** photographs at the same magnification of tissue on the FS slide (shown as an inset) and of the tissue left in the block, the one for permanent section having an area 20× larger than that for FS (H&E both at × 40), **b, c** tissue that is folded or rolled is likely to obscure fungus so it is not visible on H&E (× 250), but fungus can stain and be seen in the thick necrotic stroma on PASF-fs (× 200)

Errors in interpretation can be reduced by adhering to morphologic criteria consistent with fungus along with attention to the shapes of spaces and to any patterns they may form. Differentiating these mimics and pitfalls from fungus, however, becomes a balancing act between caution and over interpretation. But an easily correctable error is in assuring proper sampling of the BX, which was not always done in our cases where some were under sampled for FS DX while others were ‘cut away’ leaving little tissue for final DX. However, technical problems cannot always be prevented as necrotic tissue on sectioning frequently tears, folds or rolls. Fungus can be obscured in the thick areas on H&E where the hyphae can be identified on PASF-fs stain (Fig. 5).

In our study the false NEG rate for the FS DX in AIFRS was 19%, which correlates with that in the literature ranging from 12.5 to 27.3% [8, 10, 11]. This rate, however, does not truly reflect the difficulty posed in its FS DX. Including the 6 false POS with the 12 false NEG diagnoses in our study, there were 18 false diagnoses for 63 true POS diagnoses (1 false for every 3.5 true POS), a rate not generally acceptable for a pathologic DX. In at risk patients compromised immunologically and often hematologically where surgical procedures impose a further risk, pathologists sometimes add modifying comments making their diagnoses “conditional”, which may not provide the surgeon with clear support for decisions in clinically ambiguous situations. Four false NEG cases are shown as examples of the difficulties encountered in their FS DX (Supplemental Figs. 2–5).

Considering the ‘substandard’ rate of correct diagnoses, alternate methods for the DX of AIFRS were considered. Utilizing a rapid processor would enable quality formalin fixed H&E and GMS stained slides for DX within as little as 6 h from the time of BX [10]. Yet, this would require the commitment of technical staff, the availability of a rapid processor for STAT use, and sufficient proximity to the patient to avoid transportation delays. Our laboratory, like many, is designed with the batch processing required for its efficiency and productivity, and specimens for rapid processing must be received in the morning to be accommodated in the laboratory routine. In our cohort, 91% (43/47) of the FS diagnoses were performed when no histology staff was available for the full 6 h technical time required for rapid processing.

For a diagnostic method less rapid than FS to be feasible, patients amenable to the delay would need to be identified. In one study, the specimens from 12/41 (29%) of patients who were biopsied without suspicious endoscopic findings were POS on FS DX, demonstrating a lack of sensitivity in identifying the disease in its early stage. The effect of delay in DX resulted in an adverse outcome in our patients with false negative diagnoses. Surgical treatment in 6 was delayed from 24 to 48 h with more extensive debridement required, and the potential to prevent death was lost in one where necrosis progressed extensively into the orbit and surrounding tissues [16]. Added to any delay in DX is the process of preparation for surgery with these patients frequently needing platelet and sometimes red cell transfusions.

Improving the accuracy of the FS DX of AIFRS as the method most able to meet the clinical need was addressed initially by utilizing knowledge of the pathology, the patterns of necrosis and pitfalls in identifying fungus, which in our study increased the true POS diagnoses from 81 to 94% in the reviewed DX. But this incremental improvement requires an experience with an uncommon disease that is not generally available. The ability to identify fungus consistently in necrotic tissue was then recognized as required for the improvement in diagnostic accuracy. A Diff Quik (modified Wright Giemsa) stain has been used and microwave techniques for GMS attempted without sufficient success. Our histology laboratory modified the Periodic Acid Schiff’s Reaction stain for fungus for use on FSs and utilized it in the real time DX on four biopsies for suspected AIFRS with fungus staining well where it was present. This staining ability was confirmed when it was applied retrospectively over the existing original H&E stained slides and prospectively for DX on new cases in the final phase of the study.

**Fig. 6:**
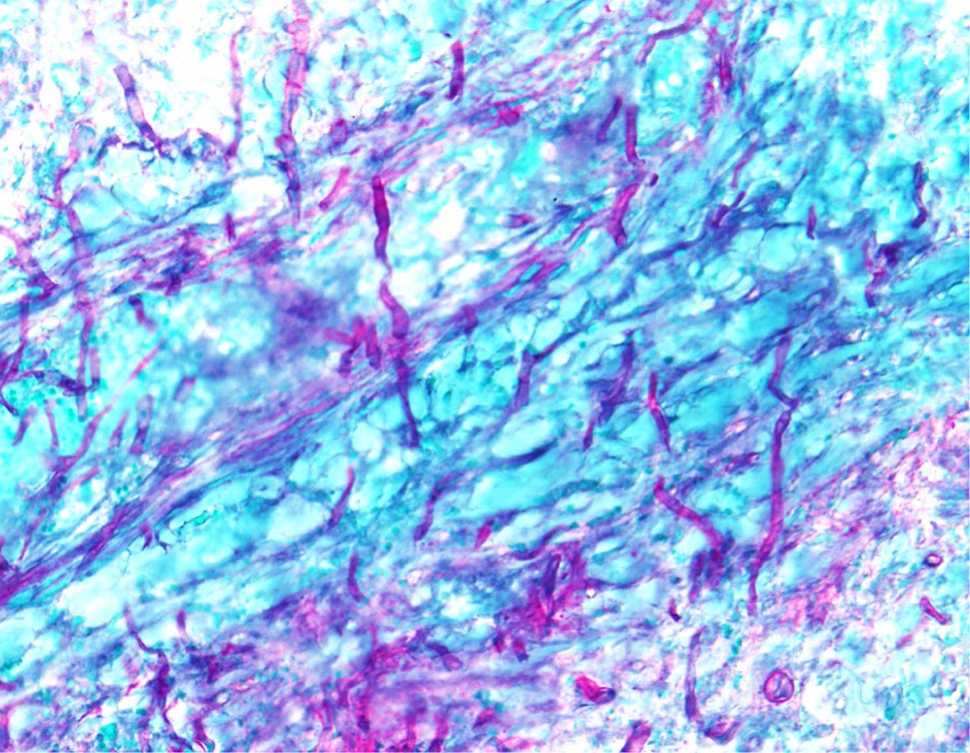
PASF-fs stain: pink staining fungus is contrasted well by the green counter stain when not stained over H&E (PASF-fs × 200)

The photographic images we present of the PASF-fs stained retrospectively demonstrating fungus not visible on the original FSs, generally have a red to purplish/gray background from the underlying H&E rather than the usual green counterstain, which contrasts well with the pink/red staining fungus. On prospective staining with PASF-fs, fungus is shown counterstained green without being masked by underlying H&E (Fig. 6). Photographic images in this article representing the H&E and corresponding PASF-fs are of the paired FS slides prepared routinely at two levels of the same BX, matched to the same tissue focus as much as possible. All photographs except for the GMS are of FSs with their inherent artifacts and variations in quality, compromised technically at times by the tissues being necrotic.

The new PASF-fs procedure we propose as an addition in the FS is simple, requires only 25 min, is easily performed by the technical staff in the gross room and amenable for use 24 h a day, being done by our resident staff nights and weekends. The PASF-fs stain in demonstrating its ability to identify fungus not otherwise visible, addresses the major cause of false NEG diagnoses, offering the potential improvement needed in the rapid accurate FS DX of AIFRS. This new staining method for fungus in FS, however, has not been validated in prospective studies.

## Conclusions

Necrosis with fungus invading tissues constitutes the DX of AIFRS. Its recognition in the patterns seen in the disease is an essential component of the DX and even when fungus in not identified, has a high correlation with the presence of AIFRS. The novel use of a rapid PASF stain modified for FS provides a major improvement in the identification of fungus, and represents the crucial outcome of this study. Its ability to improve the accuracy of FS DX as demonstrated retrospectively and prospectively in its initial use, offers promise for a more effective intervention in this fulminant potentially fatal disease. The initial small prospective series awaits further studies for true validation.

## Electronic supplementary material

Below is the link to the electronic supplementary material.

Supplemental Online Resource File (DOCX 17 KB)

Supplementary Figures (DOCX 24601 KB)
