# Supplementary material for: Acute Invasive Fungal Rhinosinusitis: Frozen Section Histomorphology and Diagnosis with PAS Stain
Source: Head Neck Pathol. 2018 Sep 12;13(3):318–26. doi: 10.1007/s12105-018-0965-8 (PMC6684546; doi:10.1007/s12105-018-0965-8)
Supplement: Supplementary file 1 — Supplemental Online Resource File (DOCX 17 KB) [file 12105_2018_965_MOESM1_ESM.docx]

**Acute Invasive Fungal Rhinosinusitis: Frozen Section**

**Histomorphology and Diagnosis with PAS Stain**

**Journal Name:** Head and Neck Pathology

Henry Crist^1^ • Max Hennessy^3^ • Jacob Hodos^1^ • Johnathan McGinn^2^ • Bartholomew White^1^ • Sakeena Payne^2^ • Joshua I Warrick^1^

^1^Department of Pathology, The Pennsylvania State University, College of Medicine, Hershey,

PA

^2^Department of Surgery, Division of Otolaryngology – Head and Neck Surgery, The

Pennsylvania State University, College of Medicine, Hershey, PA

***Corresponding Author:***

Henry S. Crist, M.D.

The Pennsylvania State University

College of Medicine

Department of Pathology

500 University Drive

Hershey, PA 17033-0850

Phone #: 717-531-1668

Fax #: 717-531-5021

E-mail: hcrist@pennstatehealth.psu.edu

**Supplemental File: Periodic Acid Schiff’s Reaction for Fungi for Frozen Tissue (PASF-fs)**

**Purpose:** The demonstration of fungi in fresh frozen tissue.

**Principle:** The principle is similar to that of a PAS procedure for Carbohydrates. Polysaccharides present in fungal cell walls are oxidized by the periodic acid to aldehydes. The aldehydes react with Schiff’s reagent to yield rose colored fungi.

**Fixative:** Fresh frozen tissue

**Equipment:** Coplin jars (three additional to those for the H&E staining procedure)

**Technique:** Cut fresh frozen tissue at 8-10 µm

**Quality Control:** Any tissue with known fungi

**Reagents:**

0.5% Periodic Acid

Periodic acid….....0.5 g

Distilled water…..100 ml

Schiff’s Reagent

Commercially bought (Thermo Scientific Ref# 88017)

Glacial Acetic Acid

Commercially bought (J.T. Baker Ref# 9508-02)

Light Green Stock Solution

Light Green SF Yellowish……0.2 g

Distilled Water………………….100 ml

Acetic Acid………………………..0.2 ml

Light Green Working

Light Green Stock Solution……..10 ml

Distilled Water………………………..50 ml

**Procedure:**

1. Quick fix slide with one dip in 95% Ethanol.
2. Wash in 2 changes of Distilled Water.
3. Submerge slide in 0.5% Periodic Acid for 10 minutes.
4. Wash in 2 changes of Distilled Water.
5. Submerge slide in Schiff’s reagent for 15 minutes.
6. Wash in tap water for 10 minutes.
7. Counterstain in Working Light Green Solution for 30 seconds to 1 minute.
8. Rinse quickly in tap water.
9. Dehydrate in 2 changes of 95% ethanol, 2 changes of 100% ethanol.
10. Clear in 2 changes of Xylene.
11. Coverslip with synthetic resin(Permount)

**Results:**

**Fungi…..Rose**

**Background……..Green**

**Notes:**

1. Most of the reagents listed can be bought commercially in a kit if desired.
2. All times are set up for preference of staining intensity.

The staining differs from the PASF on slides from paraffin blocks in elimination of the deparaffinizing step and shortening the staining time
