# Supplementary material for: Acute Invasive Fungal Rhinosinusitis: Frozen Section Histomorphology and Diagnosis with PAS Stain
Source: Head Neck Pathol. 2018 Sep 12;13(3):318–26. doi: 10.1007/s12105-018-0965-8 (PMC6684546; doi:10.1007/s12105-018-0965-8)

**Supplementary Material**

**Supplemental Figure 1**. Mimics of Fungus: **a** Aspergillus, the diagnosis is not a problem when fungus stains on H&E or when hyphae are abundant, but spaces outlined by eosin when they are few and scattered may be misinterpreted in an attempt not to fail in identifying the disease. **b** *Fibrin strands* simulating fungal walls, **c & d** *Collagen fibers* are the most common mimic, followed by **e** *vessel walls,* **f** rarely the *walls of squamous cells*, note their thin nuclei (**a-f** all H&E 400x), **g** PAS stains basement membranes of vessel walls, and **h** smooth muscle fibers and can stain mimicking fugal walls (both PASF-fs at 400x).


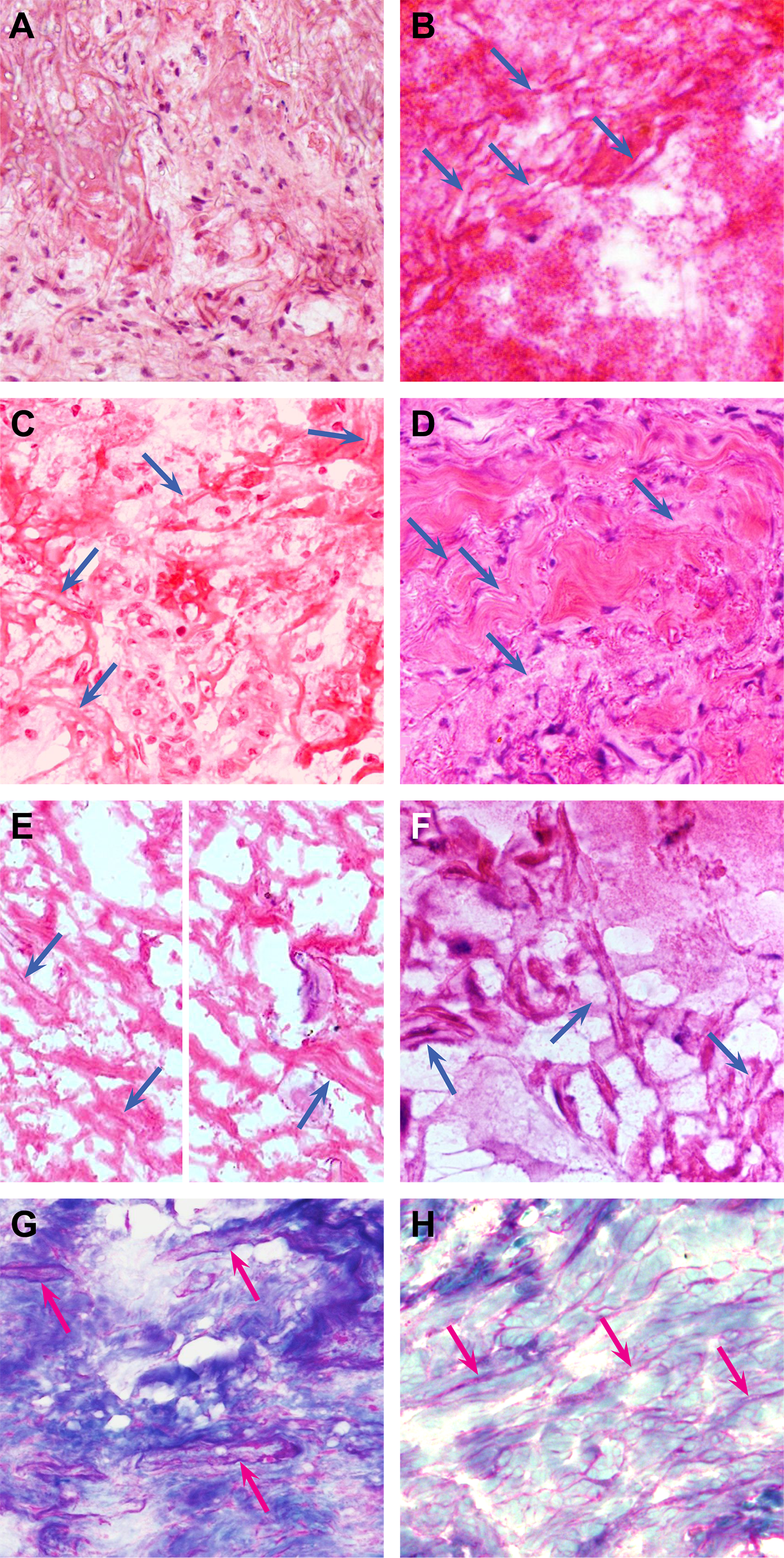


**Supplemental Figure 2.** False Negative Case: The fungus obscured by the dense necrotic tissue in this tiny ulcer could not be identified on H&E but Aspergillus was evident on PASF-fs **(a & b** H&E 40x & 300x **c** PASF-fs stain 400x).


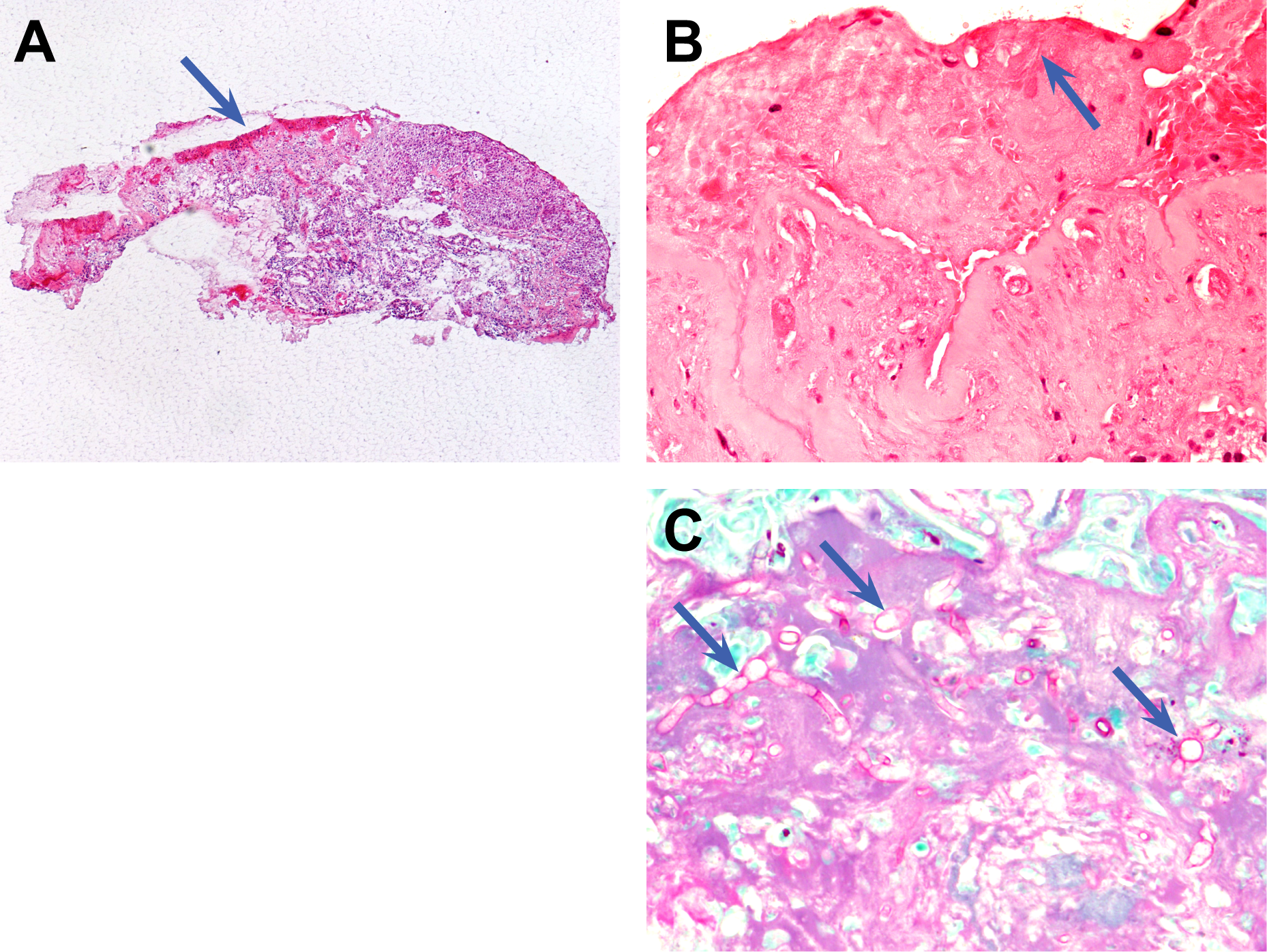


**Supplemental Figure 3.** False Negative Case: The fungus obscured and not recognizable on H&E in this biopsy with ulceration and extensive inflammation contained Aspergillus in submucosal vascular channels and stroma on PASF-fs (**a & b** H&E 40x & 300x **c** PASF-fs stain 400x).


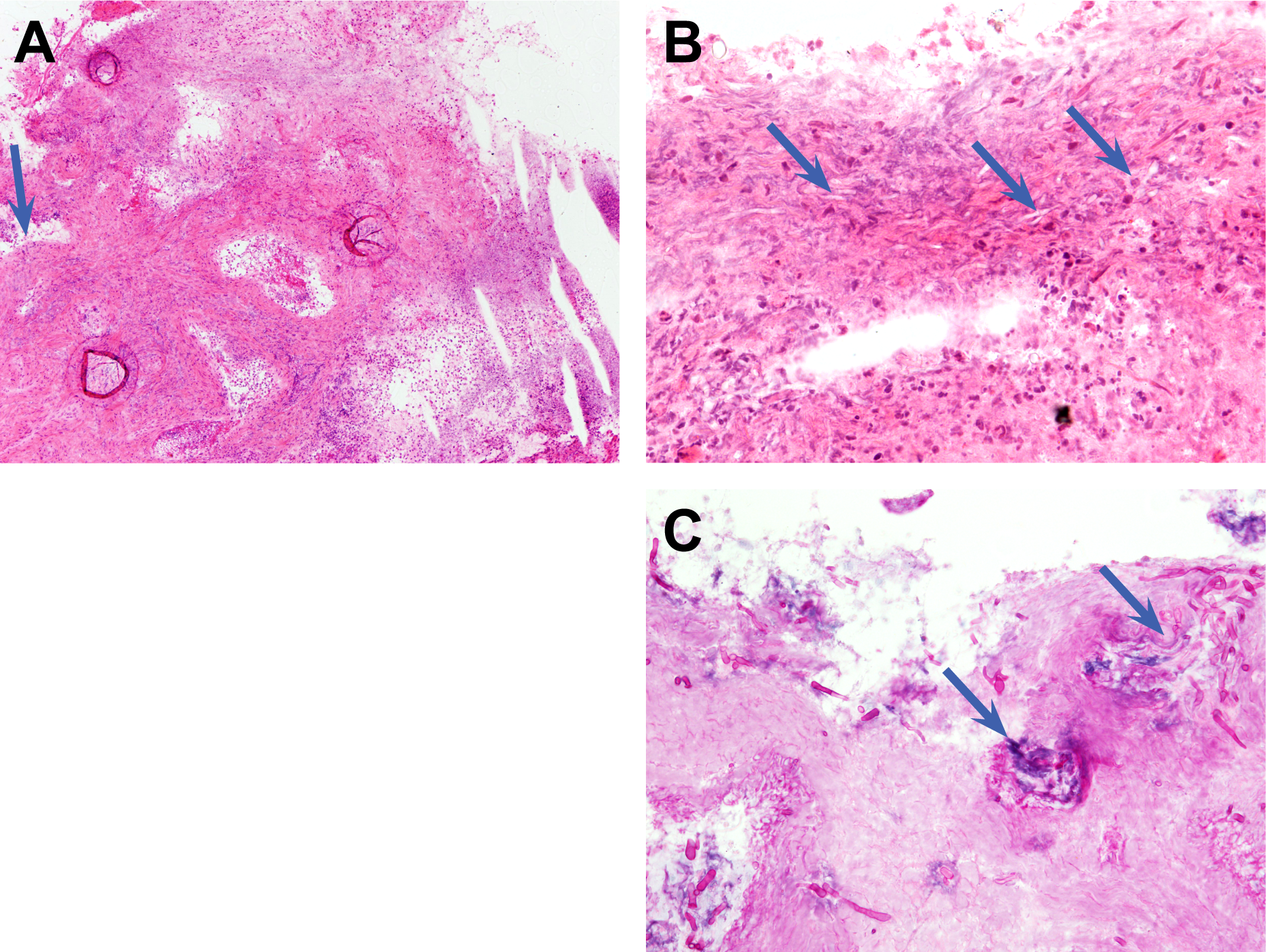


**Supplemental Figure 4.** False Negative Case: No fungus was recognized on this biopsy done at night, which was fragmented and torn with a small focus of necrosis but a few hyphae were present in a separate part so the frozen section cut for PASF-fs was not stained until the next morning. It showed Alternaria sp in the small area of necrosis identified by the arrow. The green counterstained background contrasts to the reddish/purple hue of the other images which were stained retrospectively over H&E (**a & b** H&E 40x & 250x **c** PASF-fs stain 400x).


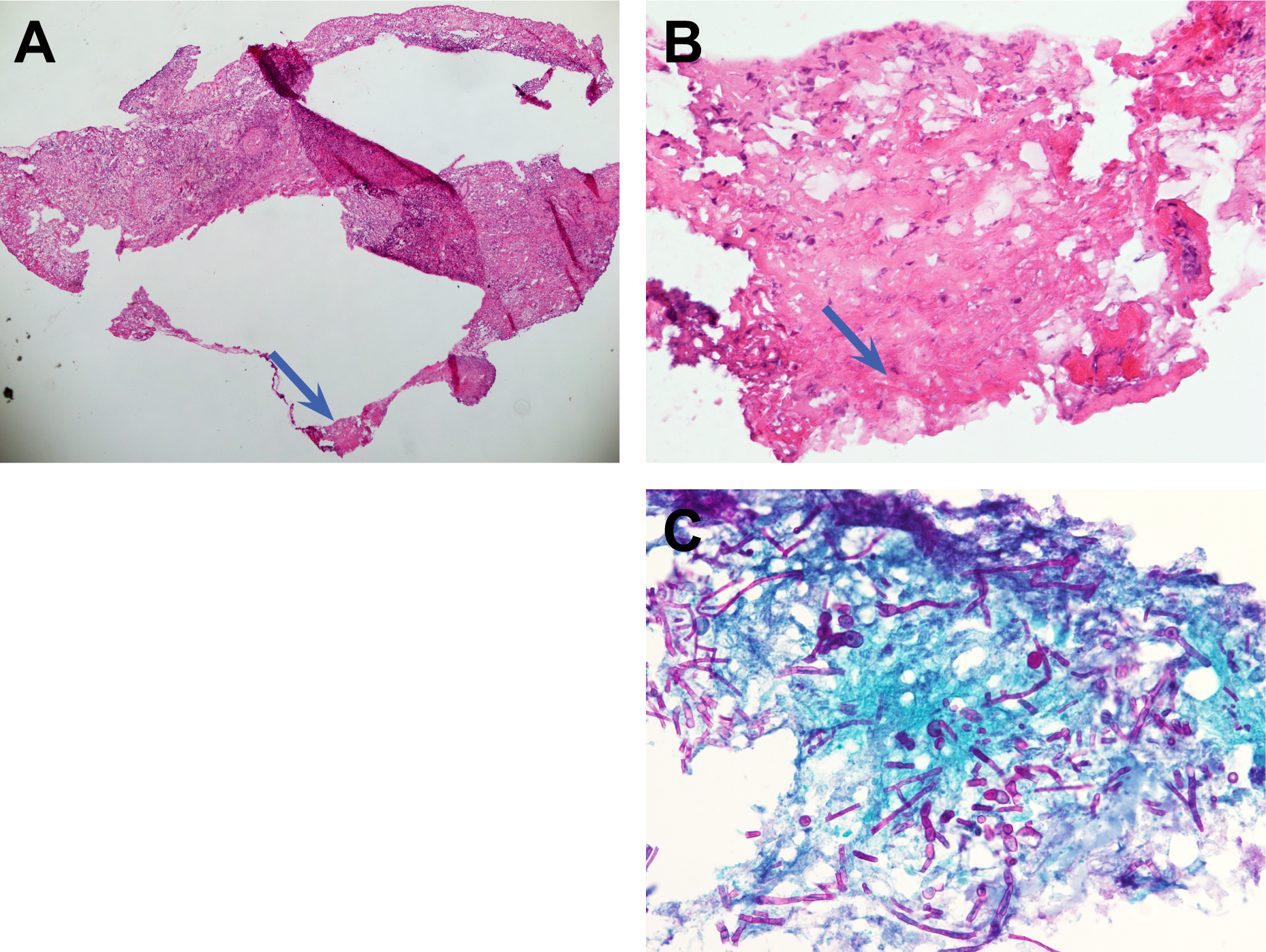


**Supplemental Figure 5.** False Negative Case: There is a tiny focus of erosion/ulceration in this biopsy which is otherwise without necrosis, but several tiny adjacent vessels contain fungal thrombi not recognized on H&E but readily evident on PASF-fs indicative of very early invasion **(a & b** H&E 40x & 300x **c** PASF-fs stain 400x).


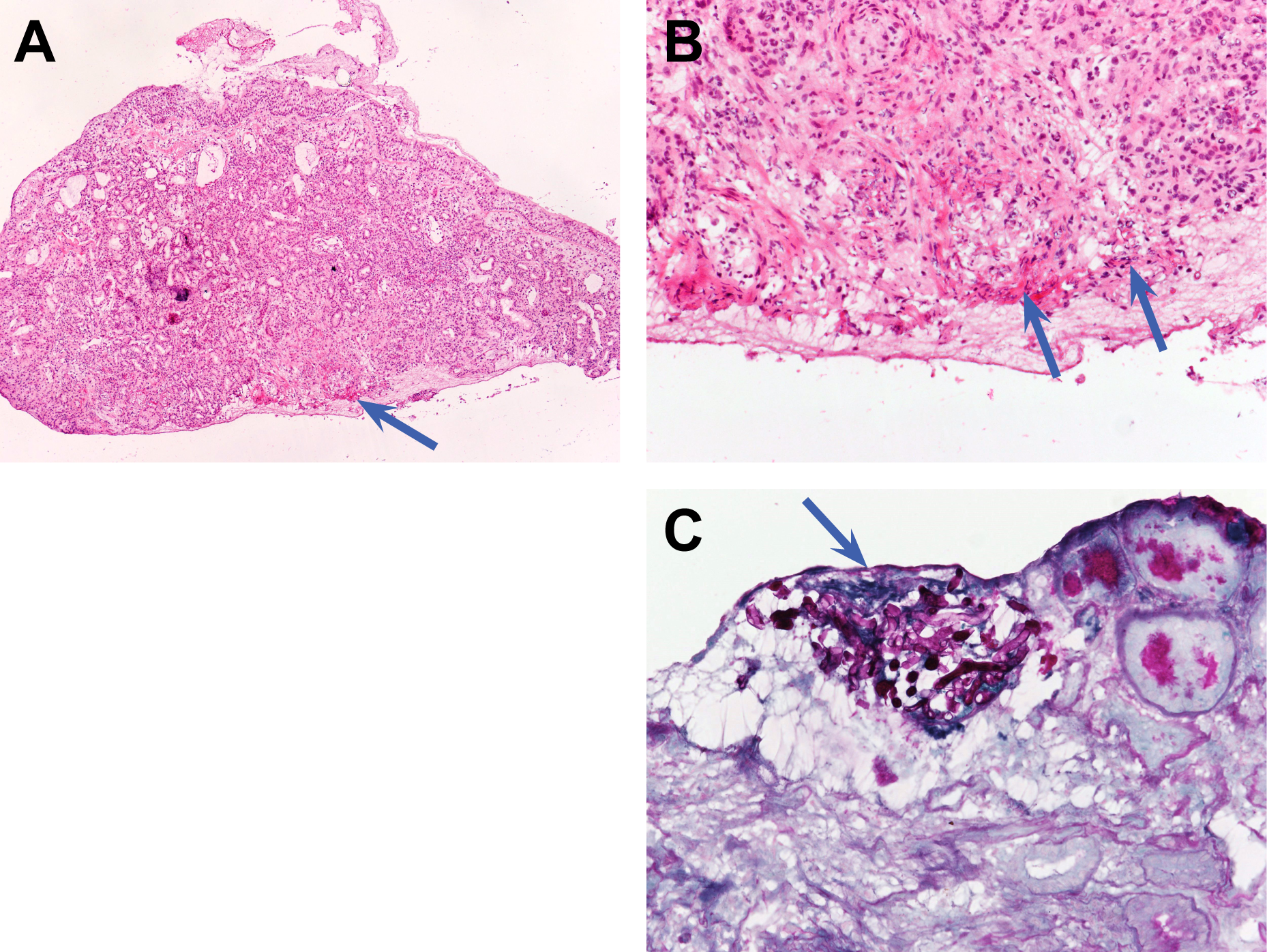

Supplement: Supplementary file 2 — Supplementary Figures (DOCX 24601 KB) [file 12105_2018_965_MOESM2_ESM.docx]
